# The psychological toll of financial volatility: compulsive habits, gambling parallels, and social policy implications

**DOI:** 10.3389/fpsyg.2026.1781407

**Published:** 2026-08-05

**Authors:** Jairo Dote-Pardo, Claudia Quezada-Baier, María Teresa Espinosa-Jaramillo

**Affiliations:** 1 Departamento de Ciencias Económicas y Administrativas, Universidad Católica de Temuco, Temuco, Chile; 2 Center of Management and Applied Economics, Universidad Católica de Temuco, Temuco, Chile; 3 Departamento de Ciencias Económicas, Administrativas y del Comercio, Universidad de las Fuerzas Armadas ESPE, Latacunga, Ecuador

**Keywords:** behavioral addictions, collective anxiety, compulsive trading, FoMO, problem gambling, risk perception

## Abstract

This mini-review synthesizes recent interdisciplinary evidence showing that financial market volatility carries psychological as well as economic costs. Across 36 studies, two domains emerge: (1) traditional market turbulence, where shocks are linked to economic anxiety, household stress transmission, and panic-selling associated with traits such as neuroticism and hyperbolic discounting; and (2) cryptocurrency and speculative asset markets, where Fear of Missing Out (FoMO), impulsivity, and herding promote short-term, gambling-like risk taking, particularly among younger or inexperienced investors. FoMO frequently mediates loss aversion and speculative engagement, while institutional participation and policy measures exhibit stabilizing, protective effects. Overall, volatility functions as a psychosocial exposure characterized by emotional contagion and behavioral reinforcement, with implications for mental health. The review calls for prevention-oriented policy integrating behavioral finance, public health, and social regulation, and for treating compulsive speculative trading as a candidate for future clinical classification, a hypothesis the current evidence base can motivate but not yet confirm.

## 1 Introduction

Financial volatility does not operate solely as an economic phenomenon but also as a psychological and behavioral stressor that shapes emotional responses, perceptions of risk, and decision-making across social groups (Simonse et al., 2024). The evidence synthesized in this mini-review indicates that periods of heightened financial turbulence, whether triggered by macroeconomic shocks, pandemics, speculative bubbles, or informational uncertainty, are systematically associated with increases in economic anxiety, panic-related investment responses, and compulsive or speculative trading behaviors. These dynamics extend beyond investors to households and broader populations, revealing the psychological toll of financial instability as a public-health concern rather than a purely market-based outcome.

Across the reviewed studies, financial shocks are shown to intensify psychological distress, perceived vulnerability, and emotional reactivity, particularly among younger adults, households exposed to income loss, and individuals with pre-existing distress trajectories. At the same time, speculative environments, especially cryptocurrency and high-risk digital asset markets, are characterized by a distinct pattern of Fear of Missing Out (FoMO), impulsivity, and gambling-like behavior, which promotes participation during volatile periods and reinforces risk-seeking actions. These findings suggest that financial volatility operates through affective, cognitive, and social contagion mechanisms that shape investment behavior and mental health outcomes simultaneously.

By framing financial volatility as a psychosocial process, rather than only a market fluctuation, this mini-review contributes to an interdisciplinary understanding of how emotional responses such as anxiety, fear, and FoMO mediate behavioral outcomes including panic selling, herding, and speculative trading. The synthesis also highlights how institutional actions, policy responses, and behavioral interventions can mitigate psychological vulnerability during crisis periods, opening a pathway toward more human-centered approaches to financial governance and investor wellbeing.

## 2 Psychological mechanisms and behavioral pathways

The findings summarized in Table 1 reveal three recurring psychological mechanisms that explain how financial shocks and speculative markets translate into behavioral risk: economic anxiety, FoMO-driven engagement, and panic-selling dynamics.

**Table 1:** Psychological responses to financial shocks, market crises, and speculative investment contexts.

No.	Core topic	Key psychological construct(s)	Main findings	Contribution to behavioral and psychological literature	Study design	Population	Market/Geographic context	Strength of inference	Model role
1	Economic anxiety during COVID-19	Economic anxiety, personality traits	Younger adults and minorities reported higher economic anxiety	Identifies psychological vulnerability profiles in early crisis stages	Cross-sectional survey	General population (younger adults, minorities)	Multi-country / early COVID-19	Moderate	Exposure
2	Investor overreaction to natural disasters	Panic reaction, climate risk perception	Investors depress prices after disasters despite fundamentals	Demonstrates emotional overreaction to physical climate shocks	Event study (archival market data)	Retail and institutional investors	Global equity markets	Moderate	Mechanism
3	Herd consumption & FoMO	FoMO, herd behavior	FoMO → brand involvement → collective consumption	Explains emotional reinforcement behind collective consumption	Cross-sectional survey	Consumers	China	Low-Moderate	Mechanism
4	Sentiment & Bitcoin volatility	FoMO, speculative excitement	Investor sentiment ↑ returns & volatility, especially post-COVID	Shows emotional trading dominates Bitcoin dynamics	Time-series econometric (market data)	Bitcoin investors	Global crypto market	Moderate	Mechanism
5	Psychological traits of Bitcoin investors	FoMO, novelty seeking, gambling tendency	Bitcoin investors show stronger impulsivity & risk-seeking	Links personality traits with speculative investment behavior	Cross-sectional survey	Bitcoin vs. share investors	South Korea	Low-Moderate	Mechanism
6	FoMO mediating herding & loss aversion	FoMO, herding bias, loss aversion	FoMO increases impact of biases on investment decisions	Establishes FoMO as a central behavioral amplifier	Cross-sectional survey (mediation model)	Retail investors	India	Moderate	Mechanism
7	COVID-19 news & Bitcoin	Investor sentiment, media exposure	Both positive & negative news influence Bitcoin price	Highlights role of information processing rather than pure FoMO	Event study / text-based market data	Crypto traders	Global	Moderate	Mechanism
8	Financial fraud & mental health	Psychological distress, quality of life	Severe financial fraud ↑ mental distress	Direct evidence linking financial harm & mental health	Cross-sectional survey	General population	Madrid, Spain	Moderate	Exposure
9	Panic expectations under currency devaluation	Market panic, collective expectations	Perceived panic selling despite weak fundamentals	Classic psychological contagion case	Theoretical / policy analysis	Currency markets	China / Hong Kong	Low	Mechanism
10	FoMO & investment intention	FoMO, behavioral biases	FoMO & intention predict investment behavior	Shows occupational context moderates risk behavior	Cross-sectional survey	Self-employed and salaried investors	India	Low-Moderate	Mechanism
11	Economic shocks & mental distress	Parental distress, child depression	Job loss ↑ anxiety, depression & family stress	Demonstrates intergenerational psychological transmission	Longitudinal cohort	Households with children	Brazil	High	Exposure
12	Covariance risk & FoMO	Fear-of-missing-out vs. flight-to-quality	FoMO episodes linked to low covariance states	Incorporates FoMO into macro-financial behavior	Asset-pricing theoretical-empirical model	Bond and equity markets	Global	Moderate	Mechanism
13	Nudging against panic selling	Endowment & IKEA effect	Portfolio ownership reduces panic selling	Evidence for protective behavioral interventions	Experimental	Retail investors	Not specified (Europe)	Moderate-High	Moderator
14	FoMO, involvement & engagement	FoMO, envy, satisfaction	Positive relationships between all constructs	Clarifies motivational mechanism behind FoMO	Cross-sectional survey	Individual investors	Turkey	Low-Moderate	Mechanism
15	Institutional behavior during crisis	Panic vs. stabilizing trading	Institutions provide liquidity, not panic sell	Psychological contrast retail vs. institutional actors	Event study (archival trading data)	Institutional investors	United States (9/11)	Moderate-High	Moderator
16	Resilience in cryptocurrency crashes	FoMO vs. panic	Limited panic — some FoMO reversal	Cryptomarkets deviate from traditional market psychology	Econometric leverage-effect analysis	Crypto investors	Global	Moderate	Moderator
17	Hyperbolic discounting & panic selling	Impulsivity, time bias	Hyperbolic discounting ↑ panic selling	Empirical connection between impatience & panic	Cross-sectional survey	Investors	Japan	Moderate	Mechanism
18	Financial FoMO & gambling risk	FoMO, speculative gambling	FoMO predicts trading & gambling harm	Emerging psychological risk in young adults	Cross-sectional survey	College students	United States	Moderate	Outcome
19	News sentiment & crypto trading	Sentiment processing, FoMO retail entry	Positive news triggers FoMO participation	Shows uninformed entry driven by emotional cues	Event study / text analytics	Crypto traders	Global	Moderate	Mechanism
20	Social policy & psychological distress	Economic distress, CMD risk	Crisis policies prevented higher CMD prevalence	Demonstrates protective policy effects on mental health	Microsimulation modeling	General population	United Kingdom	High	Moderator
21	Mood & pandemic portfolio risk	Collective mood, optimism vs. pessimism	Online searches + markets reflect global mood states	Integrates psychological mood & macro-finance	Big-data search-trend + market analysis	General public and investors	Global	Low-Moderate	Mechanism
22	FoMO & market stability	FoMO contagion	FoMO reduces system stability	Identifies systemic psychological risk mechanism	Agent-based simulation (networked minority game)	Simulated market agents	Theoretical (not empirical)	Low-Moderate	Mechanism
23	Return-volatility asymmetry in crypto	Noise trading, FoMO	Positive & negative shocks ↑ volatility	Supports behavioral rather than rational dynamic	Econometric time-series (quantile / ARDL)	Crypto markets	Global	Moderate	Mechanism
24	Neuroticism & panic selling	Personality traits, emotional instability	Neuroticism ↑ probability of panic selling	Personality strongly predicts financial behavior	Cross-sectional survey	Investors	Japan	Moderate	Mechanism
25	CSR actions under distress	Psychological distress	Social actions rewarded more during distress	Signals moral-emotional market responses	Event study (market reaction)	Firms / investors	Not specified	Low-Moderate	Moderator
26	Inequalities & mental distress	Financial stress, social isolation	Financial deterioration ↑ distress	Confirms socio-psychological vulnerability	Longitudinal cohort	Millennials	England, UK	High	Exposure
27	Life-course distress & economic shock	Chronic distress trajectories	Prior distress ↑ vulnerability to economic hardship	Demonstrates psychological path-dependency	Longitudinal cohort (birth cohorts)	Adults	United Kingdom	High	Exposure
28	Economic evaluation & mental health	Policy & psychological burden	Advocates integrating mental health into decisions	Bridges economics & psychological well-being	Conceptual / policy commentary	Not applicable	Australia	Low	Moderator
29	Investor sentiment in metaverse assets	FoMO retail dominance	Sentiment stronger in bear markets	FoMO intensifies during downturn periods	Econometric nonlinear time-series	Crypto / metaverse investors	Global	Moderate	Mechanism
30	Social interaction & investment	Social norms, peer influence, FoMO	FoMO encourages interaction & conformity	Uncovers social-relational mechanisms	Theoretical asset-pricing model	Investors	Global	Low-Moderate	Mechanism
31	Cryptocurrency speculation harm	FoMO, impulsivity, gambling	FoMO predicts financial & psychological harm	Public-health relevance	Narrative / conceptual synthesis	Crypto users	Global	Low-Moderate	Outcome
32	Determinants of crypto investment	FoMO, internal motivations	FoMO as key internal driver	Conceptual contribution	Cross-sectional survey	Retail crypto investors	Not specified	Low-Moderate	Mechanism
33	Panic selling in Malaysian market	Investor sentiment, panic	COVID-19 shocks triggered distress-based reactions	Links epidemiological & financial fear	Event study (market data)	Investors	Malaysia	Moderate	Mechanism
34	Job loss & child mental disorders	Household distress	Job loss ↑ child diagnoses	Reveals family-level emotional spillovers	Cohort / administrative data	Families with children	Brazil	High	Exposure
35	Wealth shocks & biological health	Psychological stress load	Stock shocks ↑ physiological risk markers	Demonstrates psychophysiological pathways	Longitudinal panel (biomarker data)	Individuals	Not specified	Moderate-High	Exposure
36	Spillovers & financial anxiety	Anxiety, shock contagion	Anxiety intensifies return & volatility spillovers	Emotional states propagate across markets	Econometric spillover model (time-series)	Markets / investors	Global	Moderate	Mechanism

### 2.1 Search strategy and study selection

Relevant literature was identified through searches of Web of Science (WoS) focusing on studies published between 2019 and 2025 and supplemented by selected earlier foundational studies, using combinations of the terms “financial volatility,” “economic anxiety,” “panic selling,” “FoMO,” “cryptocurrency,” “gambling,” and “behavioral addiction.” Records were screened by title and abstract for relevance to the psychological and behavioral consequences of financial market volatility, with duplicates removed prior to full-text screening. Studies were retained if they reported empirical, theoretical, or simulation-based evidence linking financial shocks or speculative trading environments to psychological or behavioral outcomes. This process yielded the 36 studies synthesized in Table 1. Given the heterogeneity of designs and the absence of a registered protocol, this manuscript is presented as a structured narrative mini-review rather than a systematic review.

### 2.2 Conceptual model: from volatility to convergent harm

**Figure 1:**
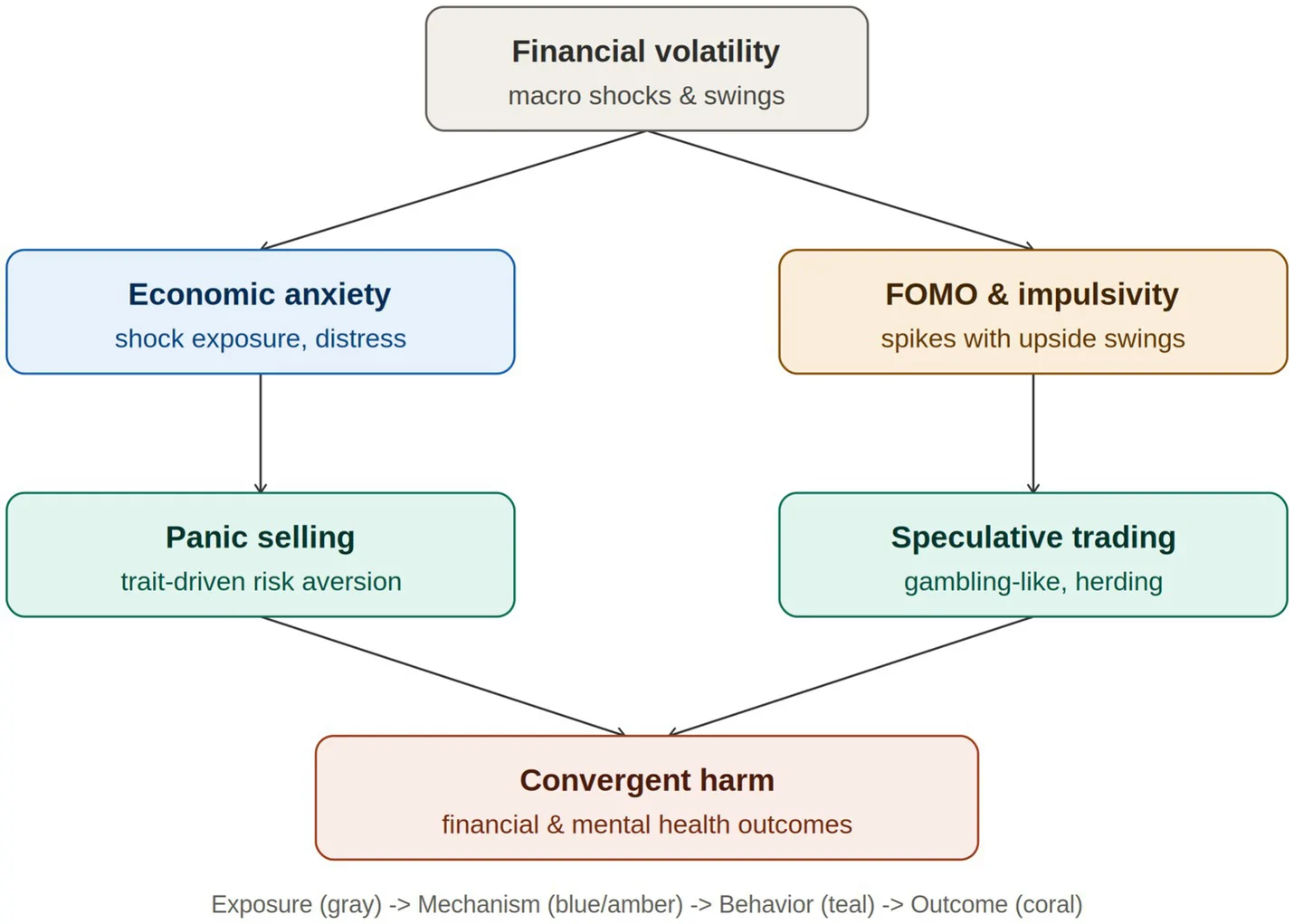
Conceptual model linking financial volatility (exposure) to economic anxiety and FoMO (mechanisms), to panic selling and speculative trading (behavioral outcomes), and to convergent financial and mental-health harm.

The studies synthesized in Table 1 can be organized into a single explanatory pathway that distinguishes exposure, psychological mechanism, behavioral response, and downstream harm (Figure 1). Financial volatility functions as the initiating exposure and bifurcates into two psychologically distinct mechanisms rather than a single undifferentiated stress response. Downward shocks and income-threatening events (job loss, fraud, market crashes) activate economic anxiety, which in turn predicts panic selling and trait-driven risk aversion. Upward and momentum-driven price swings, by contrast, activate Fear of Missing Out (FoMO) and impulsivity, which predict speculative, gambling-like trading. This is the specific sense in which FoMO intersects with financial volatility: FoMO is not a generic correlate of market turbulence but a mechanism that is selectively triggered by the upside, opportunity-signaling phase of volatility, whereas economic anxiety is selectively triggered by its downside, threat-signaling phase. Both pathways converge on a shared category of harm, financial and mental-health consequences, though they are supported by largely non-overlapping evidence bases. Table 1 classifies each study according to which link in this pathway it primarily documents (exposure, mechanism, behavioral outcome, or moderator), distinguishing studies that establish financial shocks as a risk factor from studies that establish gambling-like trading as a cause of mental-health harm.

### 2.3 Layer 1 — general financial shocks and mental health: economic anxiety and distress

Studies examining COVID-19, job loss, fraud exposure, wealth shocks, and household income deterioration consistently document increases in psychological distress, depressive and anxiety symptoms, family-level stress transmission, and in some cases physiological stress markers (Baranov et al., 2022; Fontes et al., 2024; French, 2023; Gagné and McMunn, 2023; Mann et al., 2020; Sarriá et al., 2019).

These effects are especially pronounced among younger adults, economically vulnerable households, and individuals with prior distress trajectories (Kopasker et al., 2024; Moulton et al., 2023).

Evidence from longitudinal and cohort-based research shows that pre-existing psychological vulnerability predicts stronger adverse responses to economic shocks, reinforcing a cyclical relationship between financial strain and mental health (Gagné and McMunn, 2023; Moulton et al., 2023).

### 2.4 Layer 2 — investor decision-making under market stress: panic-selling, impulsivity, and personality-based vulnerability

A second mechanism concerns panic responses during market downturns. Evidence links panic selling to: hyperbolic discounting and temporal impulsivity, emotional instability and neuroticism, and uncertainty-driven anxiety and fear contagion (Khan et al., 2024; Lal et al., 2024; Wei et al., 2000).

Simulation and network-based studies demonstrate that panic and FoMO propagate as collective behavioral dynamics, reducing market stability when social reinforcement and asymmetric expectations are present (Perras and Wagner, 2020; Park et al., 2024).

At the same time, institutional investors tend to act as stabilizers rather than panic sellers, providing liquidity during crises (Chen et al., 2019), highlighting actor-level psychological differences between retail and professional investment contexts.

### 2.5 Layer 3 — cryptocurrency and digital-asset environments: FoMO as a behavioral amplifier in speculative and retail trading contexts

Across multiple studies in cryptocurrency, retail investing, metaverse assets, and consumer herd behavior, FoMO functions as a motivational and affective driver that: amplifies herding and loss-aversion responses, strengthens participation in speculative trading, increases risk-seeking and novelty-seeking tendencies, and correlates with gambling-like harm among young adults (Argan et al., 2023; Gupta and Shrivastava, 2022; Kang et al., 2020; Kim et al., 2020; Mosbey et al., 2024; Song et al., 2024).

In several models, FoMO operates as a mediating mechanism, intensifying the influence of cognitive biases on investment decisions (Gupta and Shrivastava, 2022; Kumar et al., 2024).

These findings indicate that speculative environments are sustained not only by financial incentives but by emotionally reinforced expectations of opportunity and belonging, producing cycles of engagement even in high-volatility regimes (Güler, 2023; Gunay et al., 2024; Karim et al., 2023). These socially reinforced patterns echo evidence that peer effects and social networks shape long-term financial decisions more broadly, beyond speculative trading contexts (Mugerman et al., 2014).

Cryptomarkets represent a distinctive domain in which sentiment, news exposure, and social contagion exert strong influence on price dynamics, with positive shocks frequently triggering retail-driven FoMO participation (Brini and Lenz, 2022; Kumar Kulbhaskar and Subramaniam, 2023).

This consolidates the interpretation of speculative trading as a space where financial behavior and behavioral-addiction risk intersect (Mosbey et al., 2024; Song et al., 2024). This pattern parallels earlier behavioral-finance evidence that frequent trading is often entertainment-driven and financially costly rather than performance-driven (Barber and Odean, 2000; Dorn and Sengmueller, 2009), and that a subset of retail investors participate in equity markets for reasons closely resembling gambling motives (Kumar, 2009), which helps distinguish speculative excitement from stress-driven trading.

These three layers should not be treated as interchangeable. Evidence on FoMO-driven gambling-like behavior is drawn overwhelmingly from cryptocurrency and other high-volatility digital-asset settings and should not be assumed to generalize to financial volatility in traditional markets more broadly; conversely, findings on household economic anxiety are drawn largely from survey and cohort studies and do not, on their own, establish the addiction-like mechanisms discussed in Layer 3.

## 3 Implications, policy perspectives, and directions for future research

The integration of evidence across markets, social groups, and financial contexts supports three core implications.

Because the reviewed literature varies substantially in design and measurement, we distinguish three evidentiary tiers when interpreting these implications: (a) suggestive conceptual parallels to gambling, drawn from studies that describe similar behavioral patterns without applying clinical measures; (b) documented behavioral correlates, such as statistical associations between FoMO, impulsivity, and trading frequency; and (c) clinically validated evidence, which remains limited in this literature. Most of the crypto-specific findings summarized below fall into tiers (a) and (b) rather than (c), and this distinction should be kept in mind when weighing the policy implications that follow.

First, financial volatility constitutes a psychosocial exposure, with measurable mental-health, behavioral, and in some cases biological consequences (Baranov et al., 2022; Fontes et al., 2024; French, 2023).

This underscores the need to incorporate psychological wellbeing into economic crisis assessments and recovery strategies (Mihalopoulos et al., 2020).

Second, speculative trading, particularly in cryptocurrency and digitally mediated markets, shows preliminary evidence of features analogous to behavioral-addiction processes, including: impulsivity, FoMO-driven engagement, reward-seeking escalation, and financial harm (Kim et al., 2020; Mosbey et al., 2024; Song et al., 2024).

Third, the policy implications of this evidence are more defensible when organized around specific channels rather than presented as an undifferentiated call for regulation. Four channels are supported by the reviewed studies: (1) mental-health support during financial shocks, including employment-preserving fiscal responses (Kopasker et al., 2024) and integration of psychological burden into economic crisis evaluation (Mihalopoulos et al., 2020); (2) investor protection in high-friction retail-trading environments, informed by evidence on the performance costs of frequent trading (Barber and Odean, 2000); (3) platform design and disclosure requirements in cryptocurrency and digital-asset markets, where gambling-like harm has been most directly documented (Mosbey et al., 2024; Song et al., 2024); and (4) targeted behavioral interventions such as nudges or warnings, for which there is direct experimental evidence of reduced panic selling (Ashtiani et al., 2021).

Future research should prioritize: longitudinal anxiety–behavior feedback loops, clinical relevance of FoMO-driven trading, population-level prevention in speculative markets, and policy frameworks integrating behavioral finance, psychology, and public health.
